# Draft Genome Sequence of *Agrobacterium deltaense* Strain CNPSo 3391, Isolated from a Soybean Nodule in Mozambique

**DOI:** 10.1128/MRA.01675-18

**Published:** 2019-03-07

**Authors:** Anderson José Scherer, Jakeline Renata Marçon Delamuta, Renan Augusto Ribeiro, Amaral Machaculeha Chibeba, Stephen Kyei-Boahen, Marco Antonio Nogueira, Mariangela Hungria

**Affiliations:** a Embrapa Soja, Soil Biotechnology Laboratory, Londrina, Paraná, Brazil; b Department of Microbiology, Universidade Estadual de Londrina, Londrina, Paraná, Brazil; c CNPq, SHIS QI 1 Conjunto B, Blocos A, B, C e D, Lago Sul, Brasília, Federal District, Brazil; d International Institute of Tropical Agriculture (IITA), Nampula, Mozambique; University of Arizona

## Abstract

*Agrobacterium deltaense* strain CNPSo 3391 was isolated from a soybean nodule in Mozambique. Its genome size was estimated at 4,926,588 bp. This isolate carries several coding sequences for stress tolerance, but no identifiable nodulation or virulence genes. Possible ecological roles of bacteria isolated from legume nodules and closely related to *Agrobacterium* are discussed.

## Abstract (precis)

*Agrobacterium deltaense* strain CNPSo 3391 was isolated from a soybean nodule in Mozambique. Its genome size was estimated at 4,926,588 bp.

## ANNOUNCEMENT

For 2 decades our group has reported the isolation of agrobacteria from root nodules of soybean (Glycine max) (1–3), common bean (Phaseolus vulgaris) (4–6), and other legumes (7–9). However, the ability to reestablish nodulation with the host legume is usually not confirmed. Another example is the Agrobacterium deltaense type strain YIC4121, isolated from a root nodule of Sesbania cannabina in China; the ability to nodulate seven legumes was not confirmed (10). Here, we report the draft genome sequence of strain CNPSo 3391 (= Moz59, = 9 J1), isolated from a plant grown in Mutequelesse, Gurué District, Zambézia Province, Mozambique, showing no symptoms of N deficiency. Preliminary genetic characterization based on the 16S rRNA and three housekeeping genes positioned the strain in a *Rhizobium* (*Agrobacterium*) clade (3).

Growth conditions for CNPSo 3391 were the same as those reported for its isolation (3), and DNA extraction and paired-end sequencing on the MiSeq platform (Illumina) were performed as described before (11), resulting in 630,975,648 bp. Shotgun sequences were assembled with the A5-MiSeq pipeline (*de novo*) v.20140604 with 128-fold genome coverage assembled in 50 contigs with an *N*_50_ of 177,127 bp. The genome was estimated at 4,926,588 bp, with G+C content of 59.9 mol%, confirmed with RAST v.2.0 (12) and QUAST v.2.0 (13), using default parameters. Average nucleotide identity (ANI) (ANI calculator [14]) indicated highest similarity (97.68%) with Agrobacterium deltaense YIC4121^T^. Compared to the genomes of A. deltaense at the NCBI (strains NCPPB 1641, RV3, Zutra 3-1, and YIC4121^T^), CNPSo 3391 is slightly smaller than YIC4121^T^ (5.02 Mb), but within the same G+C range of all strains.

A total of 4,765 DNA coding sequences (CDSs) were identified in RAST (12), with 49% classified in 475 subsystems; this annotation is the public version available at GenBank. Similarly to A. deltaense YIC4121^T^, CNPSo 3391 carries no nodulation genes or *nif* and *fix* operons. However, CNPSo 3391 also carries no genes related to virulence, and we were not able to find sequences coding for *telA*, related to the speciation of some *Agrobacterium* (15). The environmental adaptability of CNPSo 3391 might be explained by genes such as 63 CDSs related to resistance to antibiotic and toxic compounds, 51 to iron acquisition and metabolism, 104 to motility and chemotaxis, and 162 to stress response.

Isolation of agrobacteria from legume root nodules seems to occur worldwide, with reports in Brazil (2, 4, 6–9), China (10), Ecuador (5), Mozambique (3), and Paraguay (1), among other countries. As these bacteria apparently do not carry nodulation genes, Yan et al. (10) suggested that they might be endophytes, but we cannot discard the hypothesis of a temporary acquisition of a symbiotic plasmid from another rhizobia. However, the role of agrobacteria in symbiosis deserves further investigation; interestingly, 3 decades ago *in vivo* results suggested that *Agrobacterium* spp. might produce extracellular “signals” that would supplement the ability of rhizobia to induce root nodulation in the host legume (16).

### Data availability.

This whole-genome shotgun project has been deposited at DDBJ/EMBL/GenBank under the GenBank accession number RRZI00000000, BioProject number PRJNA507793, BioSample number SAMN10506010, and organism number RRZI00000000; the version described in this paper is RRZI01000000.
